# Psychometric properties the of Brazilian Portuguese version of Snaith-Hamilton Pleasure Scale (SHAPS)

**DOI:** 10.47626/2237-6089-2019-0066

**Published:** 2021-02-26

**Authors:** Ana Paula Jesus-Nunes, João Paulo Barreto Borges Coroa, Felipe Coelho Argolo, Tayne de Miranda Moreira, Mychelle Morais-de-Jesus, Roberta Ferrari Marback, Fernanda S. Correia-Melo, Acioly L. T. Lacerda, Lucas C. Quarantini

**Affiliations:** 1 Programa de Pós-Graduação em Medicina e Saúde Faculdade de Medicina da Bahia Universidade Federal da Bahia SalvadorBA Brazil Programa de Pós-Graduação em Medicina e Saúde, Faculdade de Medicina da Bahia, Universidade Federal da Bahia (UFBA), Salvador, BA, Brazil.; 2 Laboratório de Neuropsicofarmacologia Hospital Universitário Professor Edgard Santos UFBA SalvadorBA Brazil Laboratório de Neuropsicofarmacologia (LANP), Serviço de Psiquiatria, Hospital Universitário Professor Edgard Santos, UFBA, Salvador, BA, Brazil.; 3 Faculdade de Medicina da Bahia UFBA SalvadorBA Brazil Faculdade de Medicina da Bahia, UFBA, Salvador, BA, Brazil.; 4 Laboratório Interdisciplinar de Neurociências Clínicas Department of Psychiatry Universidade Federal de São Paulo São PauloSP Brazil Laboratório Interdisciplinar de Neurociências Clínicas (LINC), Department of Psychiatry, Universidade Federal de São Paulo (UNIFESP), São Paulo, SP, Brazil.; 5 Departamento de Neurociências e Saúde Mental Faculdade de Medicina da Bahia UFBA SalvadorBA Brazil Departamento de Neurociências e Saúde Mental, Faculdade de Medicina da Bahia, UFBA, Salvador, BA, Brazil.

**Keywords:** SHAPS, anhedonia, psychometrics, validation studies

## Abstract

**Introduction:**

Anhedonia is defined as the reduced ability to feel pleasure and is a core symptom of various psychiatric disorders such as depression and schizophrenia. The Snaith-Hamilton Pleasure Scale (SHAPS) was developed to assess the presence of anhedonia. The objective of this study was to assess the psychometric properties of the Brazilian Portuguese version of the SHAPS.

**Methods:**

In this study, the SHAPS (14 items) was translated into Brazilian Portuguese and validated using data obtained from 228 subjects within a clinical sample. Psychometric properties were assessed using item response theory (logistic models) and classical test theory (Cronbach’s alpha). We checked for external validity using a non-parametric correlation with an independent scale: Hospital Anxiety and Depression Scale – Depression subscale (HAD-D).

**Results:**

The SHAPS presented good internal consistency, with a Cronbach’s α coefficient of 0.759 and adequacy to an IRT 1 parameter logistic (Rasch) model. The SHAPS presented significant correlation with the external measure HAD-D, with Spearman’s ρ = 0.249 (S = 1368914; p < 0.001).

**Conclusion:**

These results suggest that the Brazilian Portuguese version of the SHAPS is a reliable and valid instrument to assess hedonic tone.

## Introduction

Hedonic tone has been a subject of notable interest in the area of mental health. Anhedonia is defined as the loss or reduction of the ability to feel pleasure; it is a central feature of major depression and is present in schizophrenia.^1^ The Diagnostic and Statistical Manual of Mental Disorders, Fifth Edition (DSM-5) includes symptoms of interest or pleasure in the diagnostic criteria of major depression.^2^ Anhedonia is also a symptom of dementia and one of the non-motor symptoms of Parkinson’s disease.^3^

Research on anhedonia has contributed to the investigation of psychiatric biomarkers, mainly in depression, schizophrenia, Parkinson’s, and Alzheimer’s diseases.^4^ According to published data, it is still unclear whether anhedonia is a stable behavioral trait or a fluctuating state dependent on the severity of the disease, even after remission of the symptoms of a depressive episode.^5 , 6^

Different scales have been proposed to measure anhedonia,^7 - 9^ each with their respective limitations and disadvantages.^1^ Among the principal self-reported scales used in clinical research to evaluate anhedonia are the Snaith-Hamilton Pleasure Scale (SHAPS),^10^ the Fawcett-Clark Pleasure Capacity Scale (FCPS),^9^ the Revised Chapman Physical Anhedonia Scale (CPAS), and the Chapman Social Anhedonia Scale (CSAS).^7^ Recently, new scales have been developed to evaluate four facets of anhedonia: interest, motivation, effort, and pleasure. Examples include the Specific Loss of Interest Scale (SLIPS),^11^ the Temporal Experience of Pleasure Scale (TEPS),^12^ the Motivation and Pleasure Scale Report (MAP-SR),^13^ the Anticipatory and Consummatory Interpersonal Pleasure Scale (ACIPS),^14^ and the Dimensional Anhedonia Rating Scale (DARS).^5^ Among non-specific scales, anhedonia is typically measured using the Montgomery-Asberg Depression Rating Scale (MADRS) and the inability to feel is accessed by a single item.^10 - 15^

The SHAPS is a brief 14-item self-report measure designed to assess the presence of anhedonia and recommended for use in psychopathology research. It covers different domains of anhedonia including social interaction, food and drink, achievement, pastimes, and sensory experiences.^10^ The SHAPS demonstrated satisfactory validity and reliability in its original study, and it has shown some advantages when compared to other similar scales, e.g., lower vulnerability to cultural, sexual and age biases.^4^ In addition, the SHAPS has been validated in several languages (French,^16^ German,^17^ Japanese,^18^ Italian^4^).

Despite the recognized relevance of the SHAPS in the context of research and the clinical measuring of anhedonia,^19^ the Brazilian Portuguese version of the scale has not yet been translated or validated for use in clinical settings, meaning that the available literature is somewhat limited. The aim of this study was to translate the SHAPS into Brazilian Portuguese, use it in a clinical sample, and assess its psychometric properties.

## Methods

### Design, sample, and setting

This methodological study designed to evaluate the psychometric properties of the SHAPS was conducted at a teaching hospital affiliated with Universidade Federal da Bahia (UFBA). Patients with clinical diseases were recruited between June 10, 2010, and September 10, 2016 (n = 228). The assessments were applied to those participants who agreed to sign the informed consent form. Patients were included in the study if they met the following criteria: age ≥ 18 years and fluency/literacy in Portuguese. Patients who were illiterate and subjects who refused to sign the informed consent forms were excluded from the sample. The sample was of convenience, since all patients underwent a medical and psychological evaluation.

### Assessment questionnaires

#### Snaith-Hamilton Pleasure Scale (SHAPS)

The SHAPS is an instrument used to evaluate the experience of pleasure or the anticipation of a pleasurable experience. It is a self-reporting scale containing 14 items with four response categories: definitely agree, agree, disagree and definitely disagree. Snaith et al.^10^ proposed to recode the four categories as dichotomous (definitely agree or agree = 0; disagree or definitely disagree = 1). The higher the score, the higher the level of anhedonia.

#### The Hospital Anxiety and Depression Scale (HAD)

The HAD is a reliable instrument for screening anxiety and depression in patients with physical illnesses. It was initially developed for use in patients seen at non-psychiatric services in general hospitals. The validity of the HAD in Brazilian Portuguese was evaluated in chronic pain patients.^20^ The HAD comprises two subscales: one for anxiety and another for depression, each with 7 multiple-choice questions. The overall scores in each subscale may range from 0 to 21. The Depression subscale of the HAD (HAD-D) comprises seven questions about depressive symptoms. It is a short scale, which can be quickly filled based on information on how the patient had been feeling in the previous week.^21^

## Translation

The SHAPS was translated according to the method proposed by Wild et al.^22^ Two professional translators independently translated the original questionnaire into Brazilian Portuguese. A consensus between both translators resulted in a reconciled version, which was subsequently back-translated. The final version of the SHAPS-BR was produced to assess anhedonia in the Brazilian population and is available as online-only supplementary material.

## Ethical considerations

This study was approved by the local institutional review board (MCO-UFBA, process no. 14/2002) and was carried out in accordance with the Declaration of Helsinki. The researchers guarantee that individuals provided written consent and that the documents will be kept confidential.

## Statistical analysis

We first evaluated internal consistency using a classical test theory construct, the Cobranch’s alpha. We also fit item response theory (IRT) logistic models using one (Rasch model, 1 parameter logistic [PL]), two (2 PL) and three (3 PL) parameters, to evaluate item-wise difficulty and secondary information about the scale. We report parameters for the most adequate fit, along with information curves for the full scale and item-wise.^23 , 24^ We used Andersen’s likelihood ratio test to evaluate goodness of fit. Subsets were defined by an instrumental variable generated by separating a patient’s random protocol ID into even/odd values.

External convergent validity was assessed by checking for correlations with the independent measure (HAD-D): Spearman’s ρ correlation coefficients between SHAPS-BR and HAD-D are reported. Analyses were performed using R programming language, environment (3.6.0) and libraries *ltm* (1.1-1), *eRm* (1.0-0).^25^

## Results

### Sample characteristics

A descriptive analysis of the overall sample (n = 228) revealed that the majority of the participants were male (79.8%). The median age was 57 years (percentiles 25-75: 51.0-62.5), and the participants were predominantly married (72.12%) ( Table 1 ).

**Table 1:** Sociodemographic characteristics of sample

Variable	Patients (n = 228)
Male gender	182 (79.8)
Age, percentile 25/median/percentile 75	51.0/57.0/62.0
Education level	
Incomplete elementary school	13 (12.1)
Complete elementary school	17 (15.9)
Incomplete high school	17 (15.9)
Complete high school	31 (29.9)
Incomplete college	9 (8.4)
Complete college	20 (18.7)
Marital status	
Married	163 (72.1)
Single	27 (11.9)
Divorced	30 (13.3)
Widower	6 (2.7)
Clinical and psychiatric comorbidities	
Hepatitis B	10 (4.4)
Hepatitis C	99 (43.4)
Diabetes	60 (26.3)
Arterial hypertension	49 (21.5)
Major depressive disorder	12 (5,2)

Data presented as n (%), unless otherwise specified.

### Psychometric properties

Internal consistency, as measured by Cronbach’s alpha coefficient, was adequate (α = 0.759; n = 228; 14 items). The Andersen likelihood ratio model suggested adequate goodness of fit (likelihood ratio: 9.982; χ^2^_(12)_ = 0.618) for the Rasch 1 PL model, while multiparameter 2 PL and 3 PL models presented significant anomalies. Maximum likelihood estimation yielded large standard errors for parameters of items 3 (only for 3 PL), 4 (2 PL and 3 PL) and 12 (2 PL and 3 PL). These resulted in biased large coefficient estimates, along with odd (multi-peaked) test information profiles.

**Figure 1:**
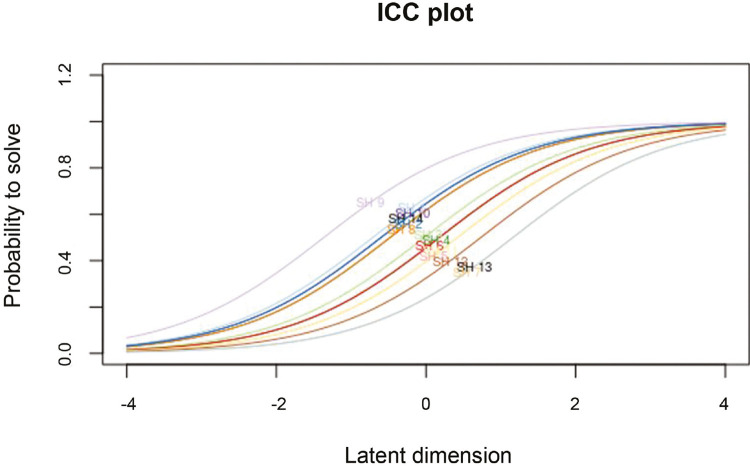
Probability of agreement with each item according to latent trait. ICC = intraclass correlation coefficient.

**Figure 2:**
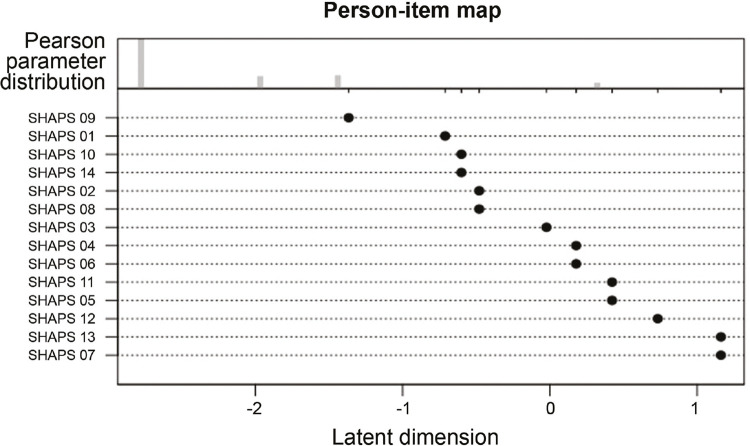
Person-item map showing parameter thresholds for each SHAPS item as well as trait distribution in the sample. SHAPS = Snaith-Hamilton Pleasure Scale.

Item characteristic curves for the 1 PL model can be observed in Figure 1 (intraclass correlation coefficient plot), showing the expected probability of a positive answer according to one’s latent trait. Figure 2 (person-item map) displays threshold scores for each item along with the distribution of estimated traits in the sample.

Table 2 presents 1 PL estimates and standard errors for each item’s difficulty (eta parameter.). Akaike information criteria (AIC), Bayesian information criterion and log-likelihood for 1, 2 and 3 PL models are shown in Table 3 .

**Table 2:** Item-wise 1 PL estimates for difficulty parameter

Item	Estimate (eta)	Standard error
1	-0.711	0.328
2	-0.480	0.354
3	-0.023	0.419
4	0.179	0.453
5	0.423	0.499
6	0.179	0.453
7	1.162	0.684
8	-0.480	0.354
9	-1.366	0.271
10	-0.601	0.340
11	0.423	0.499
12	0.733	0.568
13	1.162	0.684
14	-0.601	0.340

**Table 3:** Statistics of model adequacy

Model	AIC	BIC	Log likelihood
1 PL	1059.49	1110.93	-514.75
2 PL	860.29	956.31	-402.14
3 PL	862.07	1006.10	-389.03

AIC = Akaike information criteria; BIC = Bayesian information criterion.

**Figure 3:**
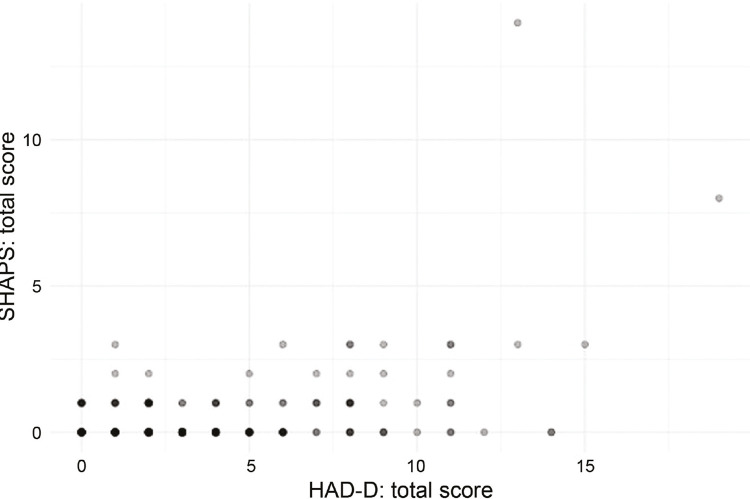
Correlation found between SHAPS and HAD-D scales using Spearman’s ρ correlation coefficients. Point opacity corresponds to frequency of observations in the point coordinate (transparency indicates low frequency). HAD-D = Hospital Anxiety and Depression Scale – Depression subscale; SHAPS = Snaith-Hamilton Pleasure Scale.

The SHAPS showed a significant correlation with HAD-D, suggesting convergent validity of the former with external measures of related constructs. Figure 3 displays a scatterplot for the positive correlation observed (Spearman’s ρ = 0.249; S = 1,368,914; p < 0.001).

## Discussion

In this study, the SHAPS was translated into Brazilian Portuguese and the validity of the resulting SHAPS-BR was examined in a clinical population. The Brazilian Portuguese version of the scale presented adequate internal consistency, with findings that were similar to those of a previous psychometric study conducted with adolescent participants (Cronbach’s alpha = 0.87). Anhedonia is a behavior pattern that can be applied to all age groups, and our result was compatible with the result of that study.^26^

Our findings support the use of the SHAPS-BR as an instrument for measuring anhedonia. Internal consistency was high and items presented overall good performance, whilst a small number of positive answers (n = 3-7) probably compromised the precision of estimates. Also, difficulties were observed for items 3, 4 and 12 in the 2 PL and 3 PL IRT models (discrimination and pseudo-guessing).

The person-item map suggests that samples presenting more severe levels of anhedonia could generate more appropriate data for investigating SHAPS-BR items, which overall presented higher thresholds in respect to our sample.

The evaluation of hedonic capacity has been useful for biomarker research in psychiatry and neurology.^4^ The SHAPS is an established tool in terms of clinical relevance in adult outpatients with major depressive disorder, as it is able to distinguish severely depressed patients from those with mild to moderate depression.^27^ Reduced levels of anhedonia may have been related to reductions in suicidal ideation in treatment-resistant patients from several clinical trials of ketamine with either major depressive disorder or bipolar disorder.^28^ Additionally, several studies have demonstrated that the SHAPS is relevant in measuring anhedonia symptomatology in medical settings, such as in patients with Parkinson’s disease^19^ and schizophrenia^17^; in liver transplant candidates, a statistically significant relationship between hepatic encephalopathy and anhedonia has been found.^29^

Previous versions of the SHAPS, validated in other languages, also demonstrated satisfactory psychometric properties: Dutch,^1^ Spanish,^30^Malay,^31^ Japanese,^18^ German,^17^ French,^16^ and Italian.^19^ A recent study of the English version of the scale in adult outpatients with major depressive disorder also demonstrated that SHAPS is a reliable and valid instrument.^27^

These findings should be treated with caution since certain limitations are present. First, the sample may have been subject to bias recruitment due to convenience sampling, and only outpatients who went to routine appointments were included. Second, the sample was composed predominantly of males and the involved patients had liver disease, so any generalization to different population profiles is limited. Nevertheless, the study comprised of a large sample of adults with clinical diseases (n = 228).

In conclusion, the results suggest that the SHAPS-BR is both reliable and valid for measuring and assessing anhedonia. The instrument can be used in clinical situations by health professionals and to conduct research in Brazil.
