# Supplementary material for: Psychometric properties the of Brazilian Portuguese version of Snaith-Hamilton Pleasure Scale (SHAPS)
Source: Trends Psychiatry Psychother. 2021 Feb 26;43(1):23–9. doi: 10.47626/2237-6089-2019-0066 (PMC7932035; doi:10.47626/2237-6089-2019-0066)
Supplement: Supplementary file 1 [file 2238-0019-trends-43-01-0023-suppl01.pdf]

http://dx.doi.org/10.47626/2237-6089-2019-0066

Online-only supplementary material

Final version of the SHAPS-BR scale (in Brazilian Portuguese)

ESCALA DE PRAZER SNAITH-HAMILTON			
Instruções: Este questionário tem como propósito medir sua capacidade de experimentar prazer nos últimos dias.<br>É importante ler cada sentença cuidadosamente.<br>Marque uma das opções para indicar o quanto você concorda ou discorda de cada uma das sentenças.			
		Concordo	Discordo
1.	Eu teria satisfação em ver meu programa de televisão ou rádio preferido		
2.	Eu teria satisfação em estar com meus familiares ou amigos		
3.	Eu sentiria prazer com meus passatempos e horários de lazer		
4.	Eu seria capaz de apreciar meu prato preferido		
5.	Eu teria satisfação em tomar um banho quente ou uma ducha refrescante		
6.	Eu teria prazer por sentir o cheiro de flores ou da brisa fresca do mar ou de pães recém-saídos do forno		
7.	Eu teria satisfação em ver outras pessoas sorrindo		
8.	Eu teria satisfação em estar com boa aparência após ter caprichado no meu visual		
9.	Eu teria satisfação em ler um livro, revista ou jornal		
10.	Eu apreciaria tomar um café ou chá ou minha bebida preferida		
11.	Eu encontraria prazer nas pequenas coisas, como por exemplo: dias bonitos de sol, um telefonema de um amigo		
12.	Eu seria capaz de apreciar uma bela paisagem ou vista		
13.	Eu sentiria prazer em ajudar outras pessoas		
14.	Eu teria satisfação em receber elogios		

Concordo = 0

Discordo = 1
